# Obituary

**Published:** 1911-12-16

**Authors:** 


### Obituary.

The death is announced as having occurred on the 6th inst., at the Royal Infirmary, Edinburgh, of Dr. Norman Campbell Young, M.B., Ch.M., son of the late Dr. Peter Young, of Edinburgh.

Surgeon-General Robert Wyatt Meadows, M.D., has died at Saltash, Cornwall, in his seventy-eighth year. He obtained a commission as Assistant Surgeon in 1854, and served in the Crimean War, being present at Inkerman and Sebastopol. He also went through the Afghan War of 1879, and was mentioned in dispatches. He became Surgeon-General in 1889. He was a well-known figure in the little town on the banks of the River Tamar, where his son, Dr. R. Thornton Meadows, is a prominent practitioner.

